# Frequency of Certain Established Risk Factors in Soft Tissue Sarcomas in Adults: A Prospective Descriptive Study of 658 Cases

**DOI:** 10.1155/2008/459386

**Published:** 2008-05-11

**Authors:** Nicolas Penel, Jessica Grosjean, Yves Marie Robin, Luc Vanseymortier, Stéphanie Clisant, Antoine Adenis

**Affiliations:** ^1^Département de Cancérologie Générale, Centre Oscar Lambret, 59020 Lille, France; ^2^Laboratoire d'Anatomopathologie, Centre Oscar Lambret, 59020 Lille, France; ^3^Département de Cancérologie Digestive et Urologique, Centre Oscar Lambret, 59020 Lille, France; ^4^Unité de Recherche Clinique, Centre Oscar Lambret, 59020 Lille, France

## Abstract

Soft tissue sarcomas are rare tumours with infrequent identified aetiological factors. Several genetic syndromes as well as previous radiation therapy and/or chronic lymphoedema have been suspected to predispose to some soft tissue sarcomas. Between January 1997 and September 2005, we carried out a prospective descriptive study to estimate the frequency of some particular etiological factors among 658 patients with soft tissue sarcomas. Sarcomas associated with a clinically identified genetic disease represent 2.8% out of all cases (95%CI: 1.5–3.8%). Most of these cases (14/19) are related to Recklinghausen neurofibromatosis. Radiation-induced sarcomas represent 3.3% out of all cases (95%CI: 1.7–5.1%). Most of these cases (9/22) are related to prior breast cancer treatment. We had observed only 1 case of Stewart-Treves syndrome. Liposarcoma, the most frequent histological subtype observed, is not associated with any particular aetiological entity. Finally, most of the adult soft tissue sarcomas are not related to any classical clinically identified genetic disease or previous radiation therapy and/or chronic lymphoedema risk factors. Frequency of underlying genetic syndrome which may predispose to soft tissue sarcomas could be higher than previously reported.

## 1. INTRODUCTION

Soft tissue sarcomas (STS) are rare tumours. Their estimated incidence is close to 3–4.5/100 000 [1, 2]. Most of these cancers had no clearly defined cause but several infrequent predisposing factors have been described, such as genetic predisposition (including mainly Recklinghausen disease and bilateral retinoblastoma) and iatrogenic factors (postirradiation sarcoma and postoperative chronic lymphoedema) [1, 2]. Several previous studies have been conducted on this topic, but have been focused on only one of these particular risk factors. There is no recent study analyzing the frequency of all these risk factors on the same cohort of patients. In order to estimate the frequency of these specific factors in adults with STS, we carried out along a 105-month period a prospective study on all new consecutive cases treated in a single institution located in Northern France area (4 millions inhabitants).

## 2. PATIENTS AND METHODS

### 2.1. Patients

We have prospectively collected some clinical characteristics of all new consecutive cases of adult (over 18 years old) (STS) treated at the Northern France Comprehensive Cancer centre (namely, Centre Oscar Lambret) between January 1997 and September 2005. Three kinds of tumours were excluded from this study, because these cases were not (Kaposi tumours, mixed mullerian tumours of uterus) or very recently (GIST) treated in our institution.

### 2.2. Data collection

The database included age at diagnosis, gender, tumour location, histological subtype, grade (according to the Fédération Nationale des Centres de Lutte Contre le Cancer System [3]), association with genetic syndrome, previous or synchronous other malignancy, postoperative lymphoedema (Stewart-Treves Syndrome), or postirradiation sarcoma. 

A pathological review or a histological diagnosis established in a reference centre is available in all cases (658). The grade is available in 384 cases (58%).

### 2.3. Definitions

The diagnosis of genetic syndrome was based on familial history criteria and clinical and phenotypic criteria [4–9]. For example, a patient meeting two or more of the following criteria can be diagnosed as suffering from Recklinghausen's neurofibromatosis: (i) neurofibromas (two or more, or one plexiform neurofibroma), (ii) “café-au-lait” macules (six or more measuring 1.5 cm in their greatest dimension), (iii) freckling in the axillary's or inguinal areas, (iv) optic glioma, (v) iris hamartomas (two or more), (vi) sphenoid dysplasia (or thinning of the cortex of the long bones), and (vii) first-degree relative [4]. The other syndromes expected were: Li-Fraumeni syndrome [5], bilateral retinoblastoma syndrome [6], Gardner syndrome or familial polyposis adenomatous [7], adult progeria [8], and Gorlin syndrome [9].

The diagnosis of radiation-induced sarcoma was based on Arlen et al. [10] criteria: (i) histological diagnosis of sarcoma, (ii) different histological diagnosis of the previous cancer, (iii) tumour in the border of radiation field, and (iv) a minimal time interval of 3 years.

### 2.4. Statistical analysis

The description of population is based on crude incidence with 95%-confidence interval for categorical parameters, median and extreme values, or mean and standard deviation for continuous parameters. The comparisons are based on Fisher exact test for categorical data and Mann-Whitney test for continuous parameters. The significance was set up at 5%.

## 3. RESULTS

### 3.1. All new cases treated between January 1997 and October 2005

The entire population included 658 cases. The sex ratio male/female was 309/349 (excluding uterus sarcoma, the sex ratio was 309/308). At diagnosis, the median age was 52 (range, 18–99). The most common histological subtypes were liposarcomas (20%), leiomyosarcomas (17%), malignant fibrous histiocytofibromas (11%), and undifferentiated sarcomas (10%). The grade was 1 in 25% of cases, 2 in 26%, and 3 in 48%. The tumour locations are listed in Table 1. The main locations were lower limbs (34%), chest wall (15%), upper limbs (13%), and retroperitoneum (10%).

### 3.2. STS associated with genetic syndrome

Nineteen patients suffered from a genetic syndrome and represented 2.8% out of all cases (IC95%: 1.5–3.8%). Most common genetic syndromes were Recklinghausen neurofibromatosis (14 cases) and bilateral retinoblastoma (2 cases). In this subpopulation, the sex ratio was 13/6 and the median age at diagnosis was 37.5 (range, 18–64). Locations, histological subtype, and grade are listed in Table 2.

 At diagnosis, these STS associated with genetic syndrome were significantly younger than the entire cohort (Median age 37.5 versus 53 years, *P* = .0016). In comparison with other cases, these patients were more frequently located on trunk (*P* = .002) and were more frequently peripheral malignant nerve sheath tumours (*P* = .005). On the contrary, liposarcomas were significantly less frequent in STS associated with genetic syndrome (*P* = .04).

### 3.3. Stewart-Treves syndrome

We had observed only one case of angiosarcoma associated with previous lymphoedema as a consequence of surgical treatment of a previous breast cancer.

### 3.4. Radiation-induced soft tissue sarcomas

Twenty two radiation-induced STSs were observed. Location, histological subtypes, and grade are listed in Table 3. The mean interval from the first cancer was 10 years (range, 3–45 years). The most common previous cancers were breast cancers (10 cases) and non-Hodgkin lymphomas (4 cases). At diagnosis, the patients were significantly older than the entire cohort (median age 66 versus 53 years, *P* = .04). In comparison with other cases, the radiation-induced were more frequently located on chest wall (*P* = .002) and were more frequently undifferentiated spindle cell sarcoma (*P* = .003) or angiosarcoma (*P* = .005). On the contrary, liposarcomas were significantly less frequent in radiation-induced sarcoma group (*P* < .001).

## 4. DISCUSSION

In this prospective study of 658 adult STS, about 6% of patients present a well-established risk factor: a genetic syndrome (2.8%) or an iatrogenic factor such as previous radiation therapy (3.3%) or a postoperative chronic lymphoedema (1 case). The characteristics of our entire group of patients are consistent literature; the sex ratio is closed to 1 [1, 2], the median age is 55 years [1], lower and upper limbs locations are the most frequent, liposarcomas and leiomyosarcoma are the most common histological subtypes (after excluding malignant fibrous histiocytofibroma), and grade 3 tumours are the most frequent [11, 12].

Twenty two cases out of 658 (3.3%, 95% CsI: 1.7–5.1%) present a radiation-induced STS. In longitudinal studies, the prevalence of radiation-induced sarcomas is very low, close to 0.14–0.20% [13–15]. After treatment by radiotherapy, the relative risk for development of STS is comprised between 8 and 50 [10, 13, 14]. As previously published [16], in our series, breast cancers and lymphomas were the most frequent previous primaries treated with radiation therapy. Radiation-induced sarcomas are more frequently STS (70%) than osseous sarcomas (30%) [16]. Malignant fibrous histiocytofibromas (16% in the Brady et al. series) and angiosarcomas (15%) are the most common histological subtype of radiation-induced STS. The liposarcomas are exceptional [16]. In the Weiss and Enzinger series, about 10% of angiosarcomas are radio-induced [17]. Radiation-induced STS are usually high-grade tumours, for example, in the Brady's series, less than 6% of radio-induced sarcomas are grade 1 [18]. The radiation-induced sarcomas are usually developed at the peripheral borders of radiation fields. The mean interval from the first cancer treatment is about 10 years (range, 2 and 67 years) [10, 13–16]. Angiosarcomas seem occur after a shorter interval (about 5 years) [10, 13–15]. The interval is also shorter in cases associated with Bilateral Retinoblastoma [6].

The Stewart-Treves syndrome is defined as the development of angiosarcoma or lymphangiosarcoma on chronic lymphoedema whatever its cause (congenital, postsurgical, or caused by filariosis,…) [18]. The Stewart-Treves syndrome is exceptional and about 300 cases are known in literature. Most of cases (168/186) are observed after axillary's clearance for breast cancer [19]. In the Connecticut Registry, 8 cases are diagnosed after the treatment of more than 41000 breast cancers [20]. The mean interval is about 10 years (4–27) for cases secondary to breast cancer treatment [18, 19, 21]. The Stewart-Treves syndrome represents about 5% of all angiosarcomas [18, 19, 21].

 In contrast to literature that describes STS are classically related to genetic syndromes in less than 1% [1] this study shows that 2.8% [1.5–3.8] of our patients suffered from a clinically-diagnosed genetic syndrome. Recklinghausen neurofibromatosis and bilateral retinoblastoma predominate. Other genetic syndromes (Li-Fraumeni syndrome, Gardner syndrome, ataxia-telangiectasia, and progeria) appear exceptional. We had no clear explanation to the present higher than previously described frequency of genetic syndrome.

 The estimated incidence of Recklinghausen Neurofibromatosis is about 1/3,000–1/5,000. Fifty percent of cases are sporadic [4, 20]. These patients had a relative risk of cancers (including STS and other sarcomas) about 4 in comparison with general population [4, 20]. Cancers are the first cause of precocious deaths in such population. About 5% of patients affected by Recklinghausen Neurofibromatosis develop malignant peripheral nerve sheath tumour (MPNST). The MPNST are usually developed on a neurofibroma [4, 22] and can be multiple [4, 22]. The male predominance is well established (sex ratio 4/1 [23]). The median age at diagnosis of STS is about 32–36, clearly inferior to age diagnosis in general population [22, 23]. Classically, about 40% of MPNST is associated with Recklinghausen Neurofibromatosis [23]. In our experience, 8 out of 24 MPNST are associated with Recklinghausen Neurofibromatosis. The prognosis of MPNST is not influenced by the presence of Recklinghausen Neurofibromatosis; the 5-year overall survival is about 40% [23]. In our study, all findings are consistent with the literature data (male predominance, young age, mainly MPNST).

 The present study presents several limitations. Firstly, our study is not exhaustive; because according to estimated incidence (3–4.4/100 000) [1, 2] of adult STS in Western countries, a total number comprised between 1140 and 1670 cases are expected in our region in the same period. In consequence, we estimate that our cohort represent between 44% and 65% of all cases. Secondly, it is a single-centre study and our results may not be directly applicable to other areas in France or abroad. The malignant nature of aggressive fibromatosis is still debated, but more recent reports suggest that a part of these tumours must be considered as a particular form of low-grade fibrosarcoma [24, 25]. Because of recent progress in histology, the proportion of the different histological subtypes must be considered with caution. For example, the “malignant fibrous histiocytofibroma” actually disappears and this diagnosis is modified into dedifferentiated liposarcoma and dedifferentiated leiomyosarcoma [26]. Moreover, the diagnosis of genetic syndromes were based on clinical criteria, a systematic genetic testing can possibly modify those results.

### 4.1. Conclusion

Most cases of adult STS (94% in our experience) are not related to well-established risk factors (radiation, genetic disease, and chronic lymphedema). Liposarcoma is the most frequent histological subtype, but it is rarely associated with genetic disease or postirradiation. New epidemiological explorations are necessary to analyze, for example, the environmental and occupational risk factors (such as arsenic, phenoxy-herbicides) and new iatrogenic factors (such as new chemotherapy agents and new techniques of radiation therapy) [27–29].

## Figures and Tables

**Table 1:** Characteristics of 658 patients with visceral and soft tissue sarcomas treated at Oscar Lambret Cancer Centre between January 1997 and September 2005. MPNST: malignant peripheral nerve sheath tumour.

Sex ratio	309 males/349 females		
Age	Median: 52 (18–99)		
	Mean: 52.4 (+/− 17.5)		

Parameter	Number of cases	Percentage	95%-CI

Liposarcoma	132	20.0	17–23
Leiomyosarcoma	113	17.0	14–20
Malignant histiocytofibroma	77	12.0	9–14
Undifferentiated sarcoma	65	9.8	7–12
Synovialosarcoma	43	6.5	4–8
Aggressive fibromatosis	32	4.8	3–6
Angiosarcoma	28	4.2	3–6
Rhabdomyosarcoma	26	4.0	2–5
MPNST	24	3.6	2–5
Others	118	18.0	15–20

Grade 1	97	25.2	21–30
Grade 2	101	26.3	22–30
Grade 3	186	48.4	43–53

Lower limbs	225	34.2	30–38
Chest wall	99	15.0	12–17
Upper limbs	86	13.0	9–14
Retroperitoneum	69	10.4	8–12
Head and neck	48	7.2	5–9
Uterus	41	6.2	4–8
Abdominal wall	40	6.0	4–8
Breast	30	4.5	3–6
Pelvis	13	1.9	1–3
Others	7	1.0	3–6

**Table 2:** Sarcomas associated with genetic syndromes.

Sex ratio	13 males/6 females		
Age	Median: 37.5 (18–64)		
	Mean: 37.5 (+/− 14)		

Parameter	Number of cases	Percentage	95%-CI

Recklinghausen disease	14	73.6	56–95
Bilateral retinoblastoma	2	10.5	0–23
Familial polypadenomatosis	1	5.2	0–14
Gorlin syndrome	1	5.2	0–14
Li-Fraumeni syndrome	1	5.2	0–14

MPNST	7	36.8	18–61
Undifferentiated sarcoma	4	15.7	2–37
Leiomyosarcoma	2	10.5	6–30
Synovialosarcoma	2	10.5	6–30
Angiosarcoma	1	5.2	0–14
Fibrosarcoma	1	5.2	0–14
Aggressive fibromatosis	1	5.2	0–14
Rhabdomyosarcoma	1	5.2	0–14

Grade 1	0	0	0–0
Grade 2	3	27.2	5–49
Grade 3	8	72.8	50–99

Chest wall	8	42.1	14–56
Lower limb	5	26.3	10–50
Head and neck	3	15.8	0–30
Abdominal wall	2	10.5	0–30
Pulmonary artery	1	5.2	0–14

**Table 3:** Radiation-induced sarcomas.

Sex ratio	5 males /17 females		
Age	Median: 66** (27–83)		
	Mean: 57 (+/− 17)		

Parameter	Number of cases	Percentage	95%-CI

Previous cancer	—	—	—
Breast cancer	10	45.0	24–66
Lymphoma	4	18.2	0–28
Cervix cancer	2	9.0	0–20
Prostate cancer	1	4.5	0–13
Bilateral retinoblastoma	1	3.5	0–13
Uterus cancer	1	4.5	0–13
Meningioma	1	4.5	0–13
Lymphoblastic acutate leukemia	1	4.5	0–13
Head and neck	1	4.5	0–13

Undifferentiated spindle cell sarcoma	11	50.0	24–66
Angiosarcoma	4	18.2	2–34
Leiomyosarcoma	2	9.0	0–20
Osteosarcoma	1	4.5	0–13
Chondrosarcoma	1	4.5	0–13
Liposarcoma	1	4.5	0–13
PNET	1	4.5	0–13
Malignant hemangioendothelioma	1	4.5	0–13

Grade 1	1	5.5	0–13
Grade 2	2	11.1	0–29
Grade 3	15	83.3	72–100

Chest wall	10	45.4	14–66
Head and neck	4	18.2	5–40
Lower limb	2	9.0	0–20
Upper limb	2	9.0	0–20
Pelvis	2	9.0	0–20
Retroperitoneum	1	4.5	0–20
Uterus	1	4.5	0–13
